# Compensation of Vestibular Function and Plasticity of Vestibular Nucleus after Unilateral Cochleostomy

**DOI:** 10.1155/2016/7287180

**Published:** 2016-01-12

**Authors:** Myung-Whan Suh, Jaihwan Hyun, Ah-Ra Lyu, Dong Woon Kim, Sung Jae Park, Jin Woong Choi, Gang Min Hur, Yong-Ho Park

**Affiliations:** ^1^Department of Otorhinolaryngology, Seoul National University Hospital, Seoul 03080, Republic of Korea; ^2^Department of Otolaryngology-Head and Neck Surgery, Dankook University Hospital, Cheonan 31116, Republic of Korea; ^3^Department of Otolaryngology-Head and Neck Surgery, College of Medicine, Chungnam National University, Daejeon 34134, Republic of Korea; ^4^Department of Anatomy, College of Medicine, Chungnam National University, Daejeon 34134, Republic of Korea; ^5^Brain Research Institute, College of Medicine, Chungnam National University, Daejeon 34134, Republic of Korea; ^6^Department of Pharmacology, College of Medicine, Chungnam National University, Daejeon 34134, Republic of Korea

## Abstract

Dizziness and vertigo frequently occur after cochlear implantation (CI) surgery, particularly during the early stages. It could recover over time but some of the patients suffered from delayed or sustained vestibular symptoms after CI. This study used rat animal models to investigate the effect of unilateral cochleostomy on the vestibular organs over time. Twenty-seven Sprague Dawley rats underwent cochleostomy to evaluate the postoperative changes in hearing threshold, gain and symmetry of the vestibular ocular response, overall balance function, number of hair cells in the crista, and the c-Fos activity in the brainstem vestibular nucleus. Loss of vestibular function was observed during the early stages, but function recovered partially over time. Histopathological findings demonstrated a mild decrease in vestibular hair cells numbers. Increased c-Fos immunoreactivity in the vestibular nucleus, observed in the early stages after cochleostomy, decreased over time. Cochleostomy is a risk factor for peripheral vestibular organ damage that can cause functional impairment in the peripheral vestibular organs. Altered vestibular nucleus activity may be associated with vestibular compensation and plasticity after unilateral cochleostomy.

## 1. Introduction

Cochlear implantation (CI) is a widely used surgical procedure for the restoration of hearing in patients with profound hearing loss. During surgery, access to the cochlea is necessary for electrode insertion. The cochlear and/or vestibular organ may be damaged during this procedure, leading to vestibular symptoms. There have been several reports of dizziness and vertigo after CI, including benign paroxysmal positional vertigo [1–3] and Meniere's disease-like symptoms [4]. Although the severity of the symptoms can vary, a large proportion of patients experience postoperative dizziness [4–6], and some of those patients experience sustained and delayed vestibular symptoms [4, 7].

The precise etiology of post-CI dizziness remains poorly understood, but it has been suggested that perilymph fistula after CI may cause disequilibrium [8]; alterations in the horizontal semicircular canal [9] and saccular function [10] may also be risk factors for postoperative vertigo. In a human temporal bone study [11], Scarpa's ganglion cell counts and peripheral vestibular hair cell densities were similar in implanted and control ears. However, cochlear hydrops and collapsed saccules were observed more frequently in implanted ears, possibly due to obstructed endolymphatic flow in the ductus reuniens or hook portion of the cochlea. Damage to the lateral cochlear wall caused by electrode insertion may also cause cochlear hydrops and delayed-onset vertigo [11]. Several authors postulate that differences in surgical procedures may also influence postoperative vertigo. Minimally traumatic or minimally invasive approaches should be preferred to decrease the risk of vestibular functional loss and vertigo [12–14].

Although several etiologies and risk factors have been suspected as causes of post-CI vertigo, impaired vestibular function is not always associated with vertigo symptoms; furthermore, the role of central compensatory mechanisms in post-CI vertigo symptoms remains to be elucidated. This study used an animal model to investigate changes over time in the peripheral vestibular organ function and in the plasticity of vestibular nucleus activity after cochleostomy.

## 2. Materials and Methods

### 2.1. Animals and Surgical Procedure

All of the animal experiments were approved by the Chungnam National University Animal Experiment Committee (CNU-00321, CNU-00500). Thirty male Sprague Dawley rats weighing 150–180 g each, with normal hearing prior to surgery, were used. There were 27 rats in the experimental group (unilateral cochleostomy; see below); the left ears underwent cochleostomy and the right ears were left untreated as a control. Different experimental animals were used at each time point (three animals at 1 and 6 hours and at 1, 3, 6, and 10 days and 9 animals at 20 days). The remaining three rats were used as normal controls in histopathological studies for brain. Before surgery, the rats were anesthetized with a combination intramuscular injection of tiletamine HCl plus zolazepam HCl (40 mg/k; Zoletil, Virbac, Carros, France) and xylazine (10 mg/kg; Rompun, Bayer Animal Health, Monheim, Germany); 0.5 mL of 1% lidocaine HCl was injected subcutaneously into the postauricular area for local anesthesia. The rats were placed in the prone position on a thermoregulated heating pad. After a retroauricular incision, the temporal bone was exposed and opened to visualize the round window membrane. Small cochleostomy was made in the bone near the round window with sharp pick on the left side. The cochleostomy site and bulla were then sealed with tissue adhesive (Durelon, 3M ESPE, Seefeld, Germany) and carboxylate cement (Durelon, 3M ESPE). The skin incision was closed in two layers.

### 2.2. Auditory Brainstem Response

To measure hearing thresholds, the auditory brainstem response (ABR) was assessed before surgery and at 1 hour and 10 and 20 days after surgery in nine rats; threshold changes were compared. TDT System-3 (Tucker Davies Technologies, Gainesville, FL, USA) hardware and software were used to obtain ABRs, with 1,000 stimulus repetitions per record. Rats were anesthetized with a combination intramuscular injection of tiletamine HCl plus zolazepam HCl and xylazine and kept warm with a heating pad during ABR recording. A subdermal (active) needle electrode was inserted at the vertex. Ground and reference electrodes were inserted subdermally in the loose skin beneath the pinnae of the contralateral and ipsilateral ears, respectively. Clicks and tone bursts, of 4 ms duration and with a rise-fall time of 1 ms at 4, 8, 16, and 32 kHz, were then presented to the right ear via an inset speculum in the external auditory meatus. Sound intensity was varied in 10 dB increments for the tone-burst sound and 5 dB increments for the click sound at near-threshold levels. The waveforms were analyzed using a custom program (BioSig RP, ver. 4.4.1; Tucker Davis Technologies, Alachua, FL, USA) with the researcher blinded to treatment group. The threshold was defined as the lowest stimulus intensity capable of evoking a wave III response > 0.2 mV. Differences in ABR thresholds were averaged across the frequency range for each cochlea to yield individual mean rises in the ABR threshold. Threshold shift was defined as the difference between the preoperative value and one of the postoperative values. A positive threshold shift indicated an elevation of the auditory threshold.

### 2.3. Vestibular Function Test

To evaluate peripheral vestibular function after cochleostomy, sinusoidal harmonic acceleration (SHA) and rotarod tests were performed before surgery and 6 hours and 1, 3, 6, 10, and 20 days after surgery, in nine randomly selected rats.

To evaluate the function of the horizontal semicircular canal, the vestibuloocular reflex (VOR) was measured using the SHA test at various rotation frequencies (0.02, 0.04, 0.08, 0.16, 0.32, and 0.64 Hz) with a peak velocity of 60 degrees/sec using an animal rotatory chair system (Jeil Hearing, Suwon, Korea). The rats were restrained on a turntable with the head firmly fixed using a bite bar. A contact-lens-type magnetic search coil was placed on the right eye for VOR measurement. Three-dimensional angular movement of the eye was recorded in the yaw, pitch, and roll planes. For further VOR analysis of the horizontal semicircular canal, only the horizontal yaw nystagmus was presented. The slow phase velocity of eye movements was extracted and plotted as a sine curve for gain and asymmetry analysis. Gain was defined as the maximum eye velocity amplitude divided by the maximum head velocity. Gain during rightward and leftward rotation was treated as a single value. Asymmetry was defined as the difference between the maximum amplitudes of slow phase velocity during rightward versus leftward rotation, divided by the sum of the two values. Normative gain and asymmetry values were based on our previous study of 16 rats (unpublished data) and were as follows: 0.17 ± 0.11 and 2.59 ± 9.8 at 0.02 Hz; 0.26 ± 0.21 and 1.33 ± 11.43 at 0.04 Hz; 0.53 ± 0.13 and 0.269 ± 10.94 at 0.08 Hz; 0.63 ± 0.17 and 1.4 ± 11.92 at 0.16 Hz; 0.59 ± 0.18 and 4.41 ± 9.29 at 0.32 Hz; and 0.51 ± 0.16 and 0.28 ± 11.95 at 0.64 Hz. Test-retest reliability was evaluated using another set of five rats: Cronbach's alpha values were 0.88 (0.02 Hz), 0.88 (0.04 Hz), 0.89 (0.08 Hz), 0.81 (0.16 Hz), 0.96 (0.32 Hz), and 0.93 (0.64 Hz). When the value was within two standard deviations of the mean, it was considered to be within the normal range.

To evaluate general balance, the rotarod test was performed using the TSE RotaRod system (RotaRod Advanced, TSE Systems, Inc., Chesterfield, MO, USA). The rotating rod was placed at a height of 1 meter to induce fear of falling. The rod was rotated at 18 rpm; the length of time in which the animal stayed on the rotating rod was measured in seconds.

### 2.4. Tissue Preparation and Immunohistochemistry

To evaluate peripheral vestibular organ hair cell survival after cochleostomy, three rats were sacrificed 20 days after surgery. Following decapitation under deep anesthesia, temporal bones were removed and the fluid spaces of the inner ear were perfused with 4% paraformaldehyde in PBS for 1 hour at room temperature. After removal of the cochlear bony walls and lateral wall tissues, the three (lateral, anterior, and posterior) semicircular canal ampullae with utricle and saccule tissues were prepared for immunostaining. For the three semicircular canal ampullae, each dissected ampulla was pretreated with 10%, 20%, and 30% sucrose in PBS and then embedded in Tissue-Tek OCT compound (Sakura Finetek Co., Tokyo, Japan) at −80°C (cryosection thickness = 20 *μ*m). For the utricles and saccules, the tissues were permeabilized with 0.3% Triton X-100 (Sigma-Aldrich Co., St. Louis, MO, USA) for 10 minutes, blocked in 5% normal goat serum (Vector Laboratories, Inc., Burlingame, CA, USA) for 30 minutes, and stained for F-actin using Alexa Fluor 488 phalloidin (Molecular Probes, Eugene, OR, USA) at a concentration of 1 : 500 for 15 minutes. After rinsing in PBS for 10 minutes, specimens were mounted on glass slides using Crystal Mount (Biomeda, Foster City, CA, USA). The hair cells of each peripheral vestibular organ were then counted. The specimens were observed using an epifluorescence microscope (Zeiss Axio Scope A1; Zeiss, Germany) with a digital camera.

**Figure 1:**
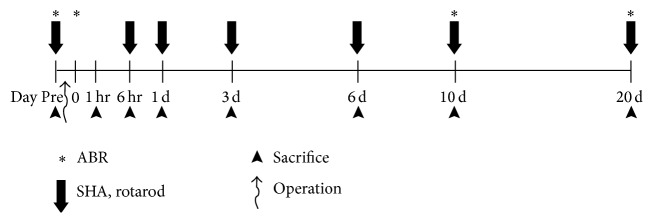
Schematic timeline of the experiments. Auditory brainstem response (ABR) thresholds were obtained before and immediately after surgery, at 10 and 20 days after surgery. The rotatory chair test (sinusoidal harmonic acceleration, SHA) was conducted prior to and at 6 hours and 1, 3, 6, 10, and 20 days after surgery. The assessment of peripheral vestibular organ hair cells was conducted 20 days after surgery. Changes in c-Fos immunoreactivity at the vestibular nucleus were evaluated at 1 and 6 hours and at 1, 3, 6, 10, and 20 days after surgery.

To observe the vestibular nucleus activity indirectly after cochleostomy, c-Fos immunoreactivities were observed in the brains of the rats which were sacrificed at 1 and 6 hours and 1, 3, 6, 10, and 20 days after surgery. After cardiac perfusion with 100 mL chilled saline, followed by perfusion with 500 mL of 0.1 mol/L phosphate buffer (pH 7.4) containing 4% paraformaldehyde, the brains were removed immediately, postfixed overnight with 4% paraformaldehyde at 4°C, and embedded in paraffin. Five-micrometer coronal brain sections of the paraffin-embedded tissue arrays were deparaffinized and rehydrated in a graded alcohol series. The antigen was retrieved with 0.01 M citrate buffer (pH 6.0) by heating the sample in a microwave vacuum histoprocessor (RHS-1, Milestone Medical Technologies, Inc., Kalamazoo, MI, USA) at a controlled final temperature of 121°C for 15 min. For immunohistochemical analyses, endogenous peroxidase activity was blocked using 0.3% hydrogen peroxide. The sections were treated with Protein Block solution (Dako) for 20 min and then incubated with specific polyclonal antisera against anti-c-Fos antibody (Santa Cruz Biotechnology, Santa Cruz, CA, USA) overnight in a humid chamber at 4°C. After washing with PBS, the tissues were exposed to biotinylated anti-rabbit IgG and streptavidin peroxidase complex (Vector Laboratories). Immunostaining was visualized with diaminobenzidine (DAB) and the specimens were mounted using Polymount (Polysciences, Inc., Warrington, PA, USA). The superior vestibular nucleus (SuVe) and magnocellular and parvicellular parts of the medial vestibular nucleus (MVeMC and MVePC) were observed in the coronal brain section (Bregma −11.60 mm). DAB-positive cells were counted at five different areas for each region in three different animals at each time point. The specimens were observed using light microscopy (Olympus BX51; Olympus, Tokyo, Japan). The timelines for all experiments are shown in Figure 1.

### 2.5. Image Processing and Statistical Analysis

Adjustments for image contrast and the superimposition of images and colorization of monochrome fluorescence images were performed using Adobe Photoshop (ver. 7.0; Adobe Systems Inc., San Jose, CA, USA). ABR threshold shift values and vestibular function test scores before and after surgery were compared using paired *t*-tests. Peripheral vestibular organ hair cell counts, in controls and after surgery, were compared using Student's *t*-test; the c-Fos immunoreactivity of the vestibular nucleus after surgery was assessed in a time series using the Kruskal-Wallis test, with Bonferroni's multiple comparison post hoc test applied if *p* < 0.05. A value of *p* < 0.05 was taken to indicate statistical significance. The InStat (ver. 3.0; GraphPad Software, La Jolla, CA, USA) and SPSS for Windows (ver. 13.0; SPSS Inc., Chicago, IL, USA) software packages were used for statistical analysis.

## 3. Results

### 3.1. ABR Threshold Shifts

**Figure 2:**
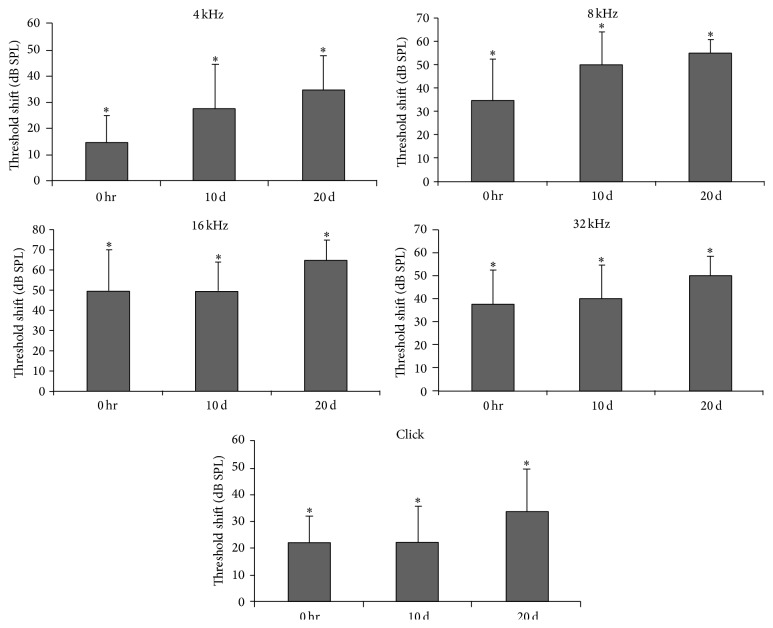
ABR threshold shifts immediately after surgery and at 10 and 20 days postoperatively. Increased ABR threshold shifts were sustained until 20 days after surgery at all measured frequencies. ^*∗*^ *p* < 0.05.

Just after cochleostomy, significant hearing threshold shifts were observed at all measured frequencies; these were sustained until 20 days after surgery (*p* < 0.05; Figure 2). These results suggest that cochlea damage or permanent hearing threshold shifts occurred after cochleostomy.

### 3.2. Vestibular Function Test

**Figure 3:**
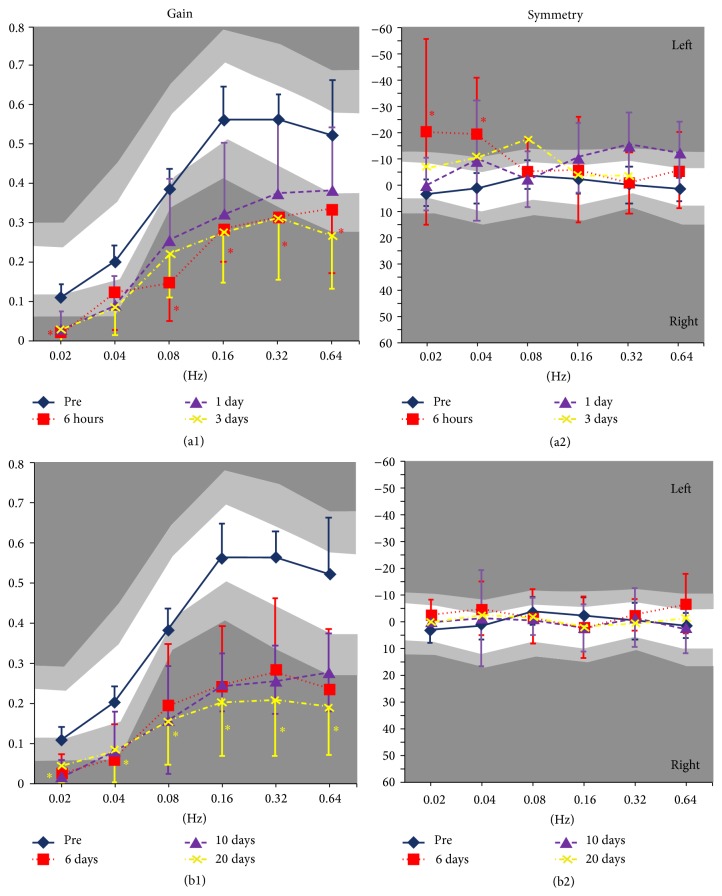
Sinusoidal harmonic acceleration (SHA) test results after cochleostomy. Before cochleostomy, gain in the SHA test was within the normal range, that is, within two standard deviations of the mean, for all rotation frequencies. However, at 6 hours after cochleostomy, the gain had decreased significantly (a1). After 6, 10, and 20 days, the gain tended to deteriorate with time (b1). Concerning symmetry, the values were within the normal range at all frequencies before cochleostomy; at 6 hours after cochleostomy, the asymmetry significantly deviated toward the side operated on at low frequencies (a2); the deviation recovered slightly after 1 and 3 days but still did not reach the normal range (0.08 Hz) (a2). After 6, 10, and 20 days, the asymmetry recovered and values were within the normal range (b2). White areas indicate normal range, light gray shadow indicates mean ± 1 SD, and dark gray shadow indicates mean ± 2 SD. ^*∗*^ *p* < 0.05.

Before cochleostomy, gain in the SHA test was within the normal range at all rotation frequencies; at 6 hours after cochleostomy, gain was significantly decreased at all frequencies (*p* < 0.05) and was below the normal range at 0.02, 0.08, 0.16, and 0.32 Hz (Figure 3(a1)). After 6, 10, and 20 days, the gain tended to deteriorate over time (Figure 3(b1)); after 20 days, it was significantly lower relative to preoperative levels at all frequencies (*p* < 0.05) and was below the normal range at 0.08, 0.16, 0.32, and 0.64 Hz (Figure 3(b1)).

Symmetry was within the normal range at all frequencies before cochleostomy; at 6 hours after cochleostomy, there was asymmetry toward the side operated on at low frequencies (i.e., 0.02 and 0.04 Hz; *p* < 0.05, Figure 3(a2)) such that vestibular function in the ear operated on was reduced relative to the ear not operated on. This deviation in symmetry recovered slightly after 1 and 3 days but remained outside the normal range at 0.08 Hz (Figure 3(b2)). After 6, 10, and 20 days, the symmetry was back within the normal range, indicating that asymmetric vestibular function was compensated for even though the gain had not recovered fully (Figure 3(b2)).

In the rotarod test, the rats were able to stay on the rod for 30.0 ± 43.3 sec before cochleostomy, which reduced to 14.9 ± 12.7 sec at 6 hours after cochleostomy. After 1, 3, 10, and 20 days, the rats were able to stay on the rod for 23.0 ± 17.2, 25.0 ± 13.2, 16.4 ± 9.1, and 23.0 ± 6.7 sec, respectively. Though not significant, the preoperative holding time was longer than the postoperative holding time, indicating worse balance after cochleostomy.

### 3.3. Histopathological Findings

#### 3.3.1. Peripheral Vestibular Hair Cell Survival

**Figure 4:**
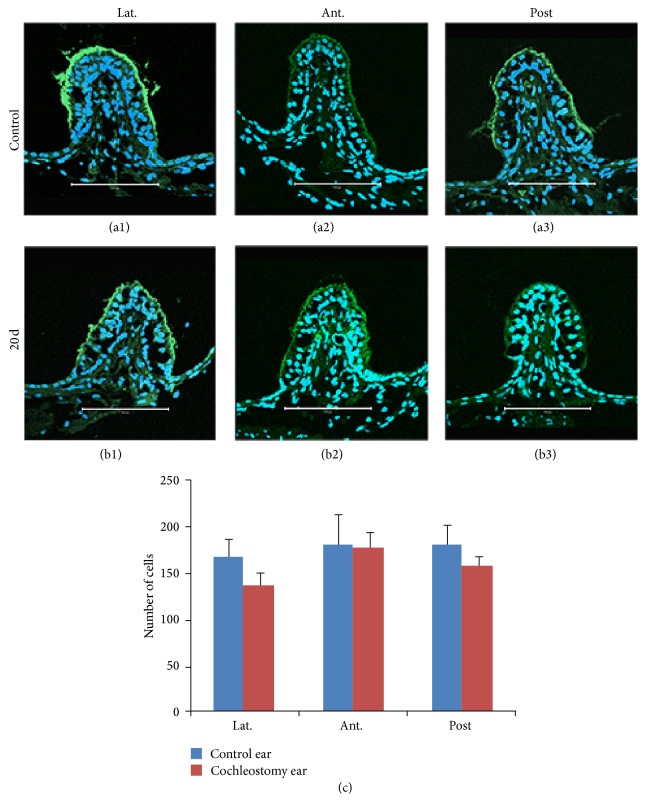
Cryosectional histopathologic findings and hair cell counts of semicircular canal ampullae in normal (a1, a2, and a3) and cochleostomy (b1, b2, and b3) ears at 20 days, stained with phalloidin (green) and DAPI (blue). Although the number of hair cells was slightly reduced in the lateral and posterior semicircular canal ampullae after cochleostomy, no significant differences in hair cell counts were observed between the cochleostomy and normal ears (c). (a1) and (b1): lateral canal ampulla; (a2) and (b2): anterior canal ampulla; (a3) and (b3): posterior canal ampulla. Scale bar = 100 *μ*m.

**Figure 5:**
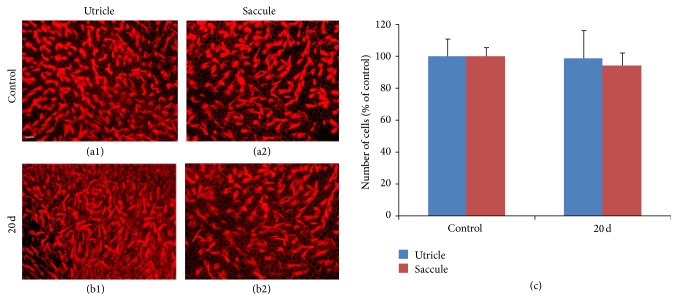
Whole mounts and hair cell counts of utricles and saccules in normal (a1 and a2) and cochleostomy (b1 and b2) ears at 20 days, stained with phalloidin (red). No significant differences in hair cell counts were observed between the cochleostomy and normal ears (c). (a1) and (b1): utricle; (a2) and (b2): saccule. Scale bar = 50 *μ*m.

The sectional images of three ampullae were similar between cochleostomy (Figures 4(b1)–4(b3)) and normal control (Figures 4(a1)–4(a3)) ears. Although a mild decrease in hair cells was observed in the lateral and posterior ampullae, there were no significant decreases in any ampulla up to 20 days after cochleostomy (Figure 4(c)). Whole mounts of the utricles and saccules after cochleostomy (Figures 5(b1) and 5(b2)) also exhibited no differences in hair cell numbers compared to normal controls (Figures 5(a1) and 5(a2)) on cell counts (Figure 5(c)).

#### 3.3.2. c-Fos Immunoreactivity in the Vestibular Nucleus

**Figure 6:**
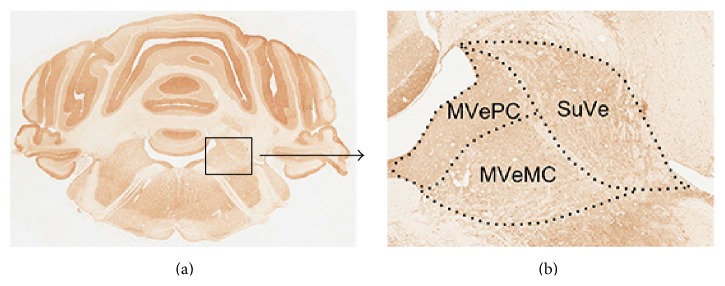
Diagram of regions of interest (SuVe, MVePC, and MVeMC) in the rat brain (Bregma −11.60 mm) stained with anti-c-Fos antibody. The box in (a) is enlarged in (b). SuVe: superior vestibular nucleus; MVeMC: magnocellular part of medial vestibular nucleus; MVePC: parvicellular part of medial vestibular nucleus.

**Figure 7:**
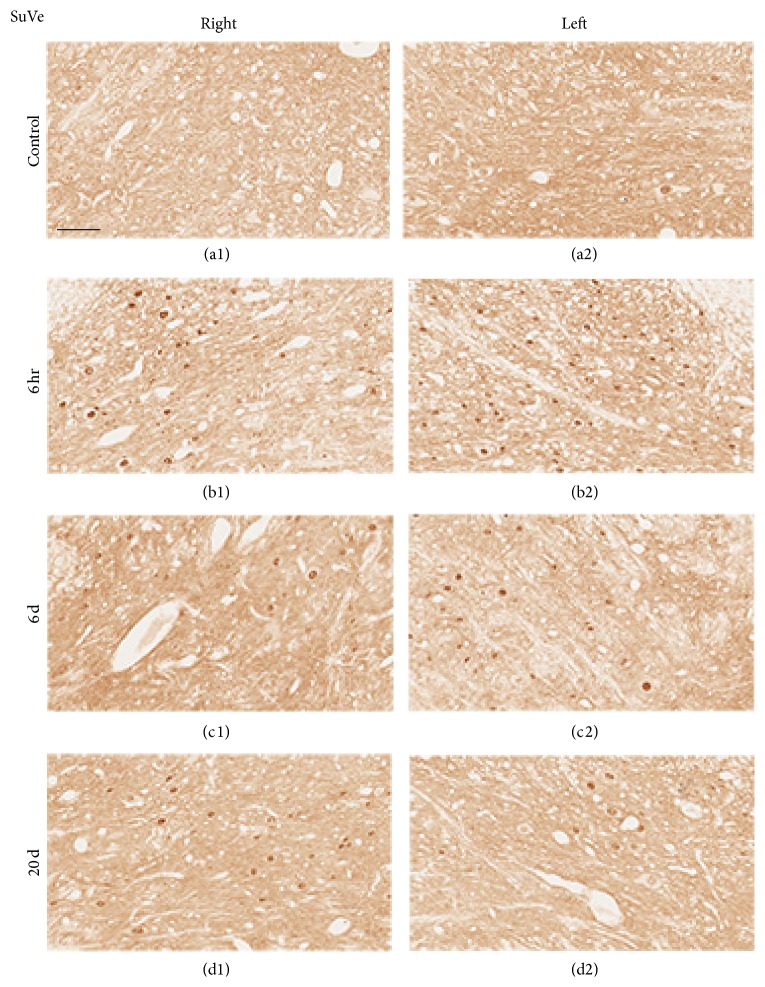
Representative photomicrographs showing c-Fos immunoreactivity in the SuVe (Bregma −11.60 mm) of rats that underwent cochleostomy at 1 and 3 hours and 6 and 20 days. Bilaterally increased c-Fos-positive cells were observed at 6 hours (b1 and b2), 6 days (c1 and c2), and 20 days (d1 and d2) relative to controls (a1 and a2). (a1), (b1), (c1), and (d1): right side; (a2), (b2), (c2), and (d2): left side. Scale bar = 40 *μ*m.

**Figure 8:**
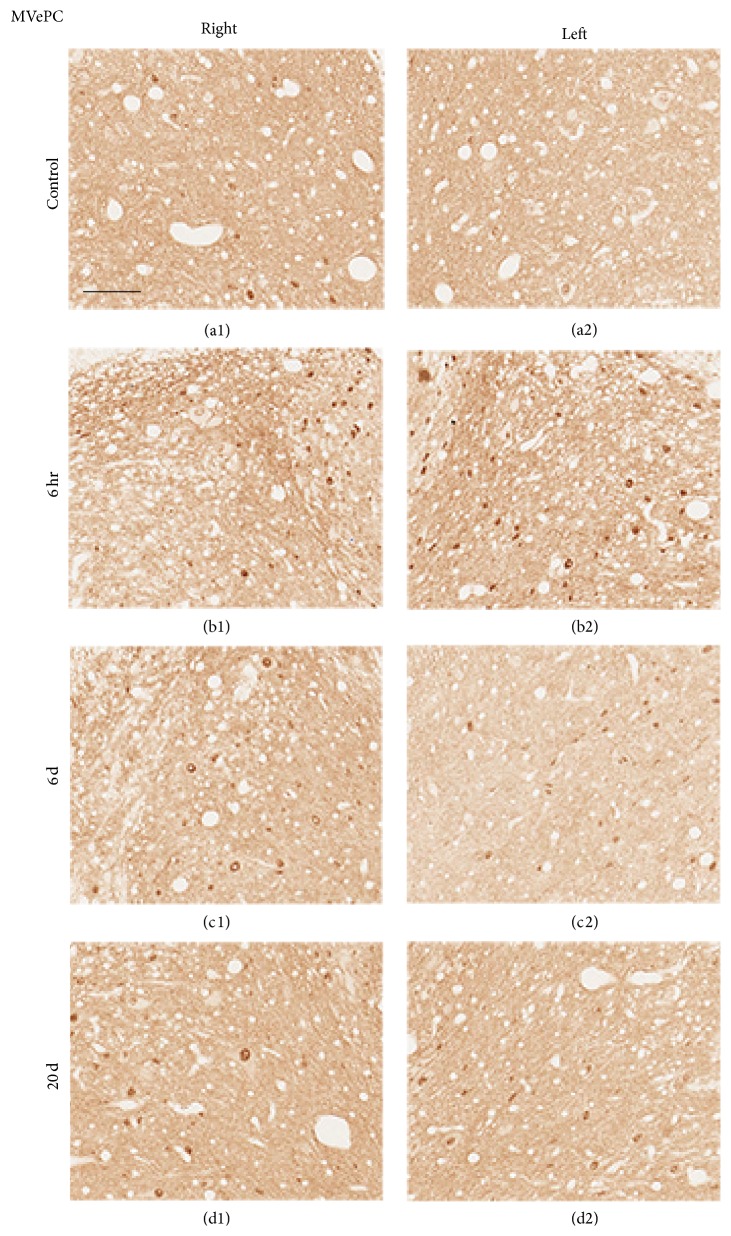
Representative photomicrographs showing c-Fos immunoreactivity in the MVePC (Bregma −11.60 mm) of rats that underwent cochleostomy at 1 and 3 hours and 6 and 20 days. Bilaterally increased c-Fos-positive cells were observed at 6 hours (b1 and b2), 6 days (c1 and c2), and 20 days (d1 and d2) compared to control (a1 and a2). (a1), (b1), (c1), and (d1): right side, (a2), (b2), (c2), and (d2): left side. Scale bar = 40 *μ*m.

**Figure 9:**
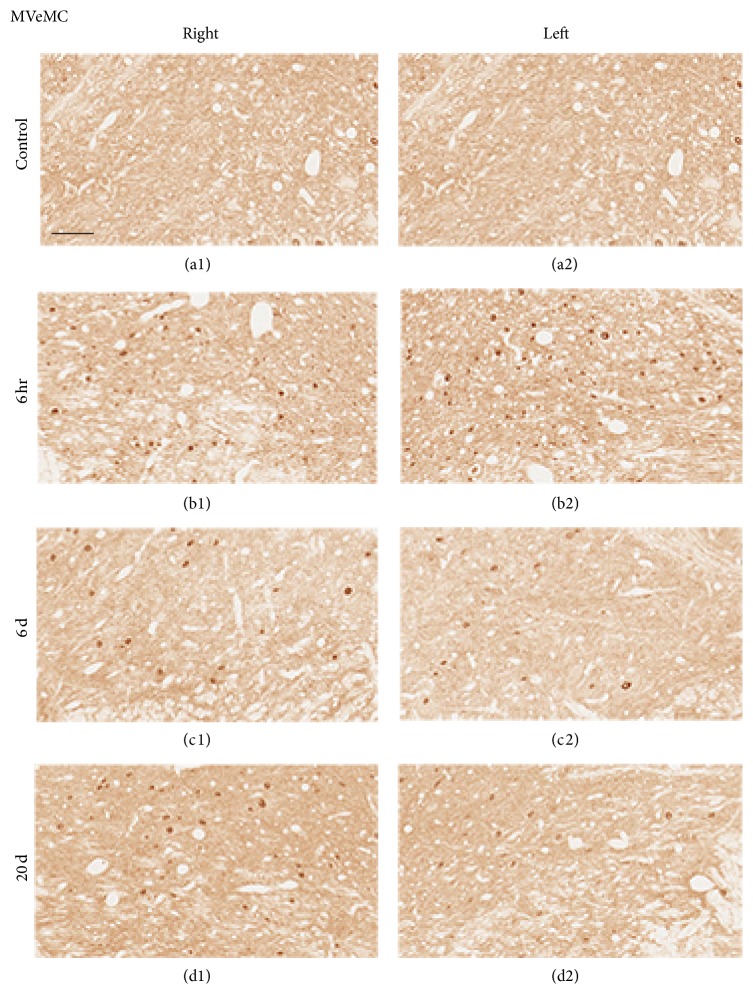
Representative photomicrographs showing c-Fos immunoreactivity in the MVeMC (Bregma −11.60 mm) of rats that underwent cochleostomy at 1 and 3 hours and 6 and 20 days. Bilaterally increased c-Fos-positive cells were observed at 6 hours (b1 and b2), 6 days (c1 and c2), and 20 days (d1 and d2) relative to controls (a1 and a2). (a1), (b1), (c1), and (d1): right side; (a2), (b2), (c2), and (d2): left side. Scale bar = 40 *μ*m.

**Figure 10:**
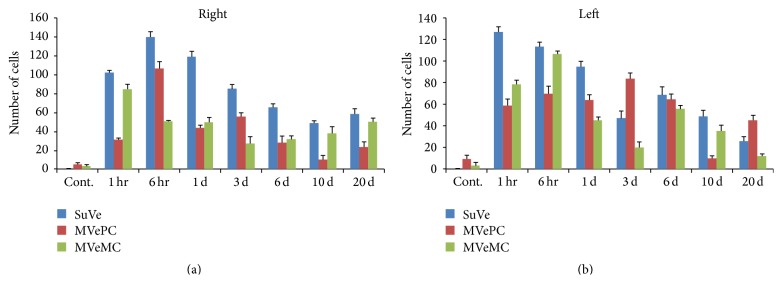
Cell counts of c-Fos-positive cells in SuVe, MVePC, and MVeMC after cochleostomy. Increased c-Fos-positive cells were observed in the bilateral vestibular nucleus, including SuVe, MVePC, and MVeMC, after cochleostomy relative to normal controls; this increased immunoreactivity was sustained until 1 day after cochleostomy and then declined slightly over time. Changes in immunoreactivity in SuVe were more marked compared to MVePC and MVeMC.

The bilateral superior vestibular nucleus (SuVe), magnocellular part of the medial vestibular nucleus (MVeMC), and parvicellular part of the medial vestibular nucleus (MVePC) were observed 11.60 mm caudal to the Bregma (Figure 6). Increased immunoreactivity was observed in the bilateral vestibular nuclei, including in SuVe (Figure 7), MVePC (Figure 8), and MVeMC (Figure 9) after cochleostomy, relative to normal controls. Increases in immunoreactivity were sustained until 1 day after cochleostomy and then declined slightly over time (Figure 10). Among the vestibular nuclei, immunoreactivity in SuVe was more marked compared to that in MVePC and MVePC. This suggests that even unilateral cochleostomy can alter bilateral vestibular nuclei activity; furthermore, peripheral vestibular disruption may be associated with changes in central vestibular tract or nucleus activity.

## 4. Discussion

Our results indicate that cochleostomy itself can significantly impair vestibular function. Low gain and asymmetry in the SHA test are typical of acute unilateral vestibular weakness. Global balance on the rotarod test also worsened after cochleostomy, though not significantly. Despite these functional changes, there was no significant difference in the hair cell counts of the peripheral vestibular organs after cochleostomy, which accords with a previous report indicating no difference in hair cell numbers between CI and contralateral control ears [11].

From a clinical perspective, the harmful effects of cochleostomy on vestibular function should be considered in patients undergoing CI. A preoperative vestibular function test could be used to evaluate the vestibular organs; if vestibular function is found to be preserved, the patient should be warned that cochleostomy and/or CI may compromise this residual function. If the patient has functioning vestibular organs in one ear only, CI on this ear may result in bilateral vestibular loss. Unilateral vestibular weakness can be treated successfully in the majority of cases, but bilateral vestibular weakness is very difficult to treat [15]. Therefore, vestibular function should be taken into account when deciding on a side for CI surgery. Because hearing preservation is an important issue in CI surgery, vestibular function-preserving “soft surgery” may be required. Furthermore, patients should be warned that they may experience vertigo after surgery due to deterioration of vestibular function.

One important difference between patients undergoing CI and our animal experiment is that vestibular function may already be partially or completely compromised in patients undergoing CI. The vestibular organs may also be impaired by the pathology that caused the hearing loss; vestibular function is abnormal in a large proportion of deaf patients even before CI [16]. If the vestibular organ is already completely nonfunctional before surgery, cochleostomy may not cause severe additional functional deterioration. However, in our experiment, all of the animals had normal hearing and vestibular function before cochleostomy; normally functioning vestibular organs may be more sensitive to potentially destructive surgery such as cochleostomy. Understanding the consequences of cochleostomy for vestibular organs is important, because in many patients vestibular function is partially or completely preserved despite hearing loss. Another difference between patients undergoing CI and our animal experiment is that a wire or another array was not inserted through the cochleostomy in this experiment. In order to replicate a condition similar to cochlear implantation, an electrode or wire inside the cochlea is essential. When designing this study, we also thought about this issue very seriously. After trying several different wires and silicon tubes, we decided not to insert any foreign material through the cochleostomy. This was because the purpose of this study was to reveal the changes in the vestibular system and not the cochlea. If the purpose of this study was to find changes inside the cochlea, despite all the shortcomings of the dummy electrode, we would have put an electrode in the cochlea. This will probably cause significant local reactions and fibrosis around the electrode. But since the vestibular organ was the target of evaluation, we thought that local reaction around the electrode was not important in this study. To mimic the current trend of minimally invasive human CI surgery, we thought that simply making cochleostomy and not inserting any nonoptimized foreign material in the cochlea would be much fairer. If we inserted something in the cochlea, we are quite sure that a larger amount of change (to be more precise, “damage”) would have resulted in the vestibule. In terms of the vestibular organs, we believe our experiment is closer to the real cochlear implant surgery (a minimally invasive surgical condition) because we did not damage the cochlea with a wire or another array.

Notably, SHA test gain gradually deteriorated after 6–20 days. After 6–20 days, it appears that additional damage not observed during our histopathological study may have developed in the peripheral vestibular end organ, thus further impairing gain. The cause of this additional damage remains unclear. Regardless of the pathogenesis, it should be noted that postoperative vestibular damage does not occur at only one time, during surgery; the harmful effects of cochleostomy on the vestibular organs appear to occur during a gradual, ongoing process that persists for at least 20 days. Certain patients who undergo CI experience immediate postoperative dizziness, but this is unlikely to persist for 20 days. The reason why patients do not experience additional dizziness despite gradual, ongoing vestibular loss remains unclear but could be due to the fact that central compensation occurs more rapidly than the gradual vestibular loss. As described in the Results, asymmetry in the SHA test returned to normal after a short period (<6 days) and remained normal thereafter despite decreasing gain. This may constitute evidence of central compensation; cochleostomy could cause ongoing, gradual deterioration of vestibular function for at least 20 days, but the central nervous system appears to be capable of compensating for this slow deterioration.

There have been several reports on peripheral vestibular organ damage after CI. In a human temporal bone study of individuals who underwent CI, peripheral vestibular organ damage, such as cochlear hydrops and saccule collapse, was observed [11]. Although we did not observe these features, hair cell counts in the semicircular canal ampullae and utricles and saccules of cochleostomy ears at 20 days did not differ significantly from normal control ears. However, there was a mild decrease in hair cells in the lateral and posterior canal ampulla. Therefore, it appears that cochleostomy itself may damage peripheral organs, including the membranous labyrinth.

The central vestibular system is more complex than the peripheral vestibular organ; it contains four vestibular nuclei (superior, lateral, medial, and inferior), plus the cerebellum and various tracts. Changes in GABA, histamine H3 receptor, and glycin have also been reported during vestibular compensation [17–21]. There has also been a recent report of c-Fos changes in the brainstem after labyrinthectomy [22], in which increased c-Fos immunoreactivity was observed in the bilateral medial vestibular nucleus (MVe), bilateral spinal vestibular nucleus, contralateral prepositus hypoglossal nucleus, and contralateral inferior olive nucleus. This change suggested that the plastic events would occur in the vestibular nucleus after unilateral labyrinthectomy or deafferentation of peripheral vestibular organ and the plasticity of spontaneous excitatory and inhibitory synaptic activity is associated with vestibular compensation [23]. These findings were observed also with functional brain imaging study [24]. In our study, we evaluated c-Fos immunoreactivity in SuVe, magnocellular part of the medial vestibular nucleus (MVeMC), and parvicellular part of the medial vestibular nucleus (MVePC) over time after cochleostomy. Increased c-Fos-positive cells were observed in the bilateral SuVe, MVeMC, and MVePC after unilateral cochleostomy, which accords with a previous report [22]; other areas, including SuVe, may also be associated with central compensation and plasticity.

In this study, we evaluated the effects of unilateral cochleostomy on the peripheral vestibular organ by measuring changes in the activity of central vestibular nuclei. Mild changes in the peripheral vestibular organs were observed during histopathological investigation; disrupted vestibular function was observed in the early stages after cochleostomy but recovered over time. Cochleostomy affects the activities of the vestibular nuclei, which may be associated with central vestibular compensation and plasticity.

## 5. Conclusions

In this study, peripheral vestibular organ damage and functional loss occurred in cochleostomy ears; this function recovered partially over time. The bilateral central vestibular nucleus was associated with unilateral cochleostomy and may also be associated with central compensation after vestibular functional loss. For cochlear implant patients, preoperative vestibular function testing may be important; less destructive or careful surgery should be considered for patients in whom the vestibular organs remain functional.
